# Enhancing cellulose functionalities by size reduction using media-mill

**DOI:** 10.1038/s41598-018-29777-w

**Published:** 2018-07-27

**Authors:** Rajni Dubey, Yon-Rui Toh, An-I Yeh

**Affiliations:** 1 Graduate Institute of Food Science and Technology, National Taiwan University, 1 Roosevelt Road, Section 4, Taipei, Taiwan; 2 Research Centre for Applied Sciences, Academia Sinica, Section 2, Nankang, Taipei, Taiwan

## Abstract

This study explored the feasibility of enhancing cellulose functionalities by using media milling to reduce the size of cellulose particles, and assayed various physicochemical and physiological properties of the resulting cellulose. Cellulose has been recognized as dietary fiber by USFDA due to its health benefits. However, its properties like low degradability, stiff texture, and insolubility in water limits its applicability in foods. Milling reduced the volume mean size of cellulose from 25.7 μm to 0.9 μm, which in turn increased the specific surface area (36.78-fold), and swelling capacity (9-fold). Conversely, a reduction in the bulk density (1.41 to 1.32 g/mL) and intrinsic viscosity (165.64 to 77.28 mL/g) were found. The milled cellulose also had significantly enhanced capacity for holding water and binding bile acids and sugars. Moreover, the size reduction also resulted in increased fermentability of cellulose into short chain fatty acids using three human fecal microflora samples. The increase in production of acetate (2880.60%), propionate (2738.52%), and butyrate (2865.89%) after fermentation of cellulose for 24 h were significantly enhanced by size reduction. With these improved characteristics, the milled cellulose might have beneficial physiological effects including laxation as well as reduced blood cholesterol and glucose attenuation.

## Introduction

Consumers’ increasing concern with healthy eating habits has intensified competitiveness among food industries to create nutritionally value-added products. Thus there has been increasing attention to dietary fiber (DF) as a healthy food component, due to its ability to reduce indigestion^1^, cholesterol, diabetes mellitus^2^, cardiovascular disorders and obesity^3^. It is also well known that *in vivo* fermentation of DF by anaerobic intestinal microbiota produces short-chain fatty acids (SCFAs), which have multiple beneficial effects on human metabolism^4^. A recent study confirmed that a fiber-deprived diet adversely impacts the gut microbiota, thereby elevating the risk of intestinal diseases^5^.

However, highly processed food with low DF content is an increasingly large portion of routine diets, and the daily intake of DF in most industrial countries is now considerably lower than the recommended adequate intake (RAI) levels^6^. Statistical analysis also shows that elderly subjects had much lower DF intake, due to declined swallowing ability with aging^7,8^. Hence, it is predictable that these phenomena might affect the future trend of chronic diseases in many developed countries.

Since cellulose has been recognized as a dietary fiber by the USFDA^9^ and the EU^10^, it has become one of the most promising commercial raw materials for food industries^11^. Stephen *et al*.^10^ pointed out that many individuals’ intake of dietary fiber does not reach recommended levels. In addition, incorporating DF into food products can impart stability to a high-fat diet, modify textural properties and improve shelf-life^12^. An effective way to support the intake of DF is to include it in processed food products. Thus economic and efficacious processing techniques are needed to expand its applicability. Size reduction techniques such as milling can produce micro- or nano-size particles of cellulose, altering their structure and surface characteristics, and consecutively changing some of the industrially important functional properties^13^. In recent years, various reduction methods such as ball milling^14^, ultrafine grinding^15,16^ and the shear and cooling milling machine (SCMM)^17^ have been applied to micronize DF. Size reduction of cellulose has been reported to increase solubility, water holding capacity (WHC)^18^, swelling capacity (SC)^19^ and fermentability^20^, thus enhancing its nutritional value. However, the data on *in vitro* physiological properties associated with improved nutritional values of milled cellulose, such as bile acid and sugar binding ability and SCFA production by fermentation of milled cellulose using gut microbiota has not been investigated in detail. Therefore, we characterize milled cellulose produced by media milling.

Since the physicochemical properties of cellulose may vary with milling methods and duration, we specifically investigated the effect of media milling on the morphology and *in-vitro* physiological functions, in addition to the cellulose physicochemical properties. This study revealed that nutritional value of cellulose could be enhanced by media milling, which could be used by food industries to improve human health through diet. The information from this study can help to promote the development of cellulose supplemented foods.

## Results and Discussion

### Particle size distribution

**Figure 1:**
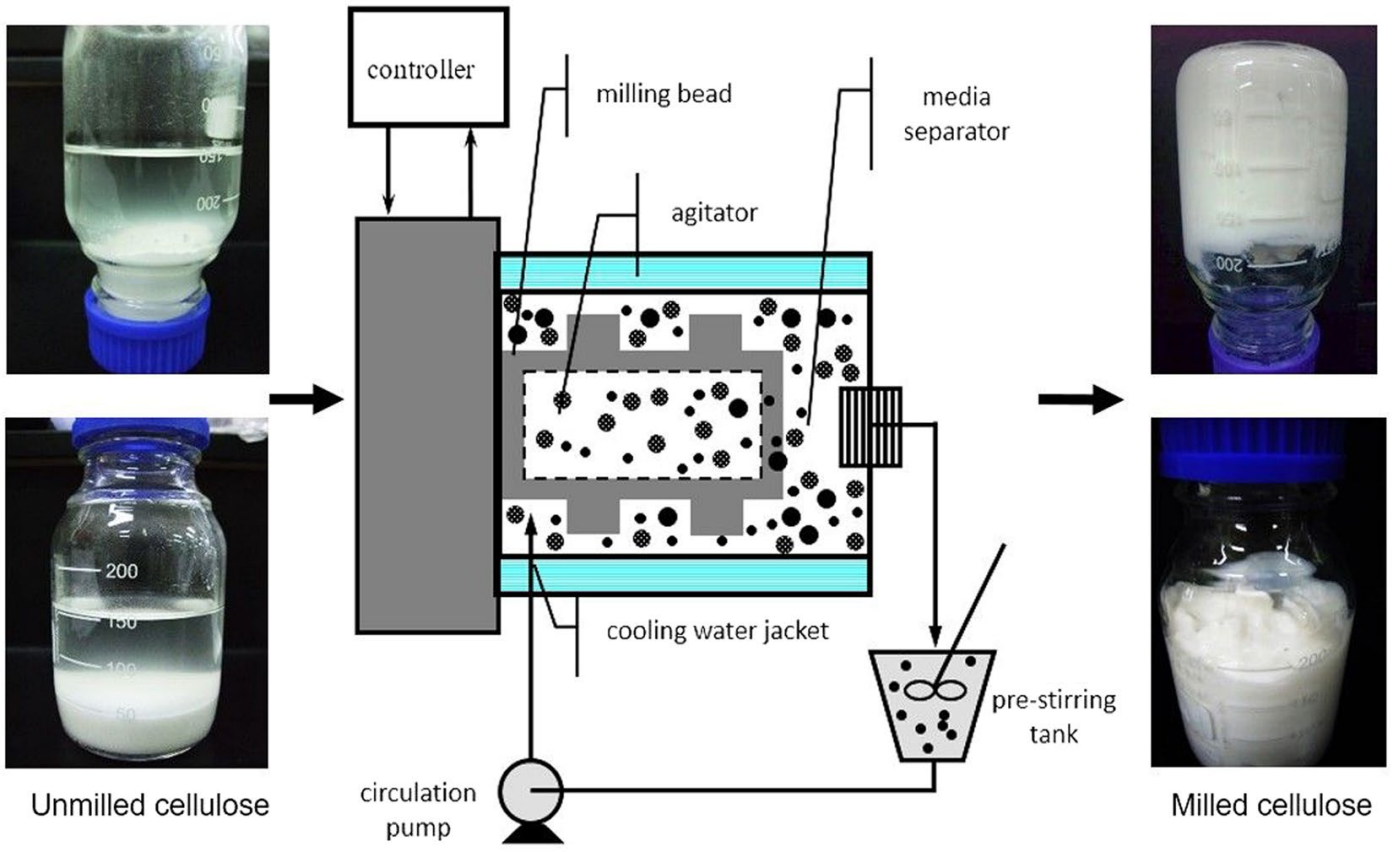
Schematic diagram of media milling machine with its feed (unmilled cellulose, 4% w/v) and product (milled cellulose) after 90 min of milling.

**Figure 2:**
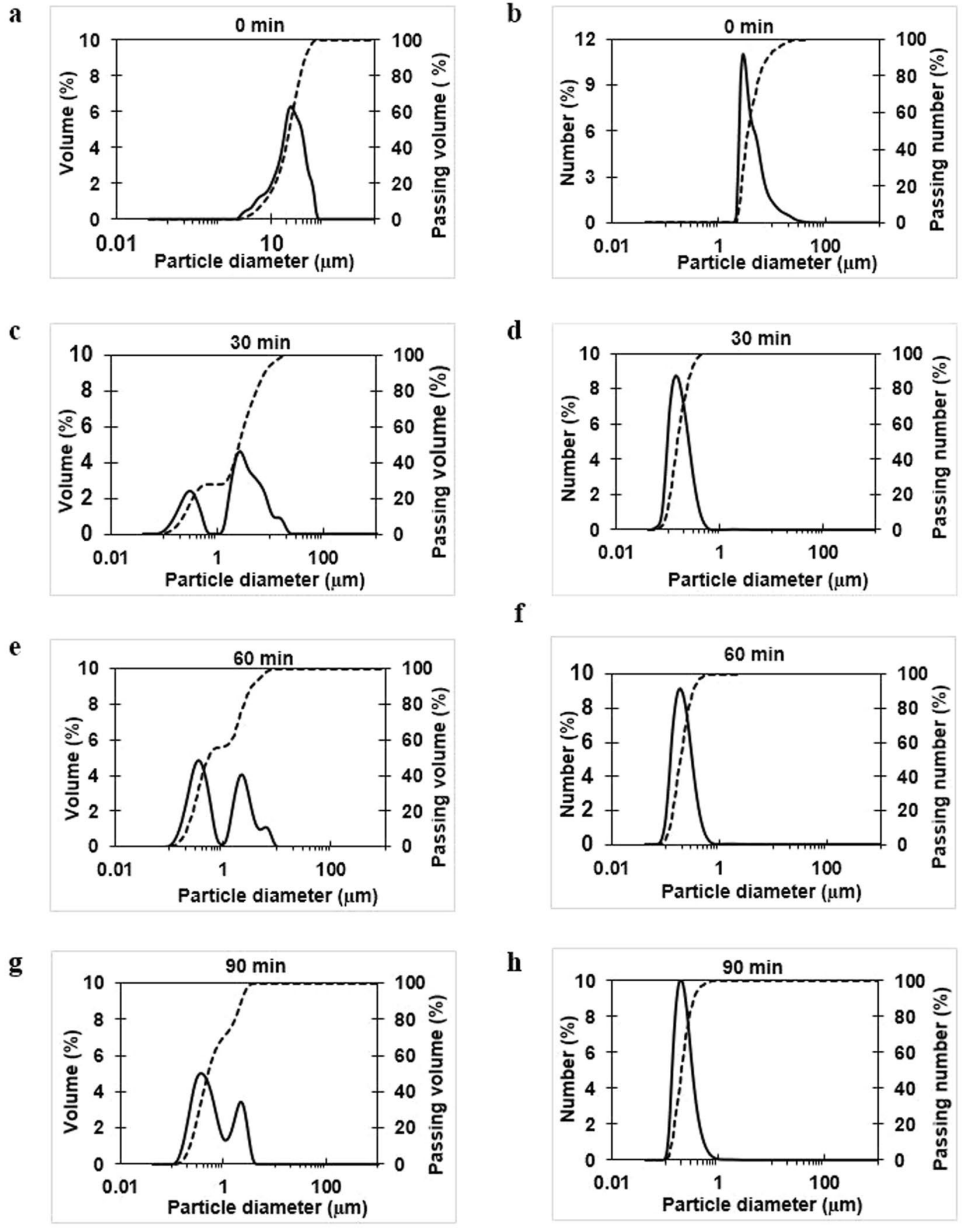
Particle size distribution of cellulose suspension before and after media milling. (**a**) Volume distribution and (**b**) number distribution of unmilled cellulose (UC) (**a**,**b**), and milled cellulose (MC) for 30 min (**c**,**d**), 60 min (**e**,**f**) and 90 min (**g**,**h**). Passing % is represented with a dotted line.

The size of the cellulose particles was reduced by media milling (Fig. 1). The particle size distribution (by volume and number) of UC and 4% MC for different milling times is shown in Fig. 2. The particles of UC exhibit a unimodal distribution (2.11 to 80.07 μm) which became bimodal after 30 min of media milling with a small and large peak at 0.1–1 μm and 1–30 μm, respectively. With prolonged milling for 60 min (Fig. 2e), the volume percentage of particles smaller than 10 μm increased; and after 90 min, particles were smaller than 1 μm. Consequently, compared to size of UC (25.7 μm), the mean volume particle size of MC was significantly reduced to 3.67 μm (−85.7%), 1.5 μm (−94.2%) and 0.92 μm (−96.4%) after 30, 60 and 90 min of milling, respectively.

Size distributions based on number showed that a higher proportion of small cellulose particles were generated by milling, which significantly reduced the number-mean particle size from 5.09 μm to 0.24 μm after 90 min (Fig. 2h). Further, the passing volume percentage of particles smaller than 1 μm was increased from 27.6% to 71.76% after 30 and 90 min. Overall, results showed that media milling effectively reduced the particle size of cellulose to sub-micron level, and this reducing effect increased with milling time. The media milling of cellulose reduced the particle size mostly to micrometer range and after milling for 90 min only 0.21% of the particles were less than 100 nm in size. The oral toxicity of media milled cellulose has been tested in our lab on male and female mice by feeding it at different doses (0.02, 0.2 and 2 g/kg/day) and results have shown that it caused no adverse clinical signs (data not shown). Moreover, the pH value of MC was found to be slightly decreased by milling, perhaps from the release of hydroxyl group due to structural degradation.

### Morphological changes in cellulose after media milling

**Figure 3:**
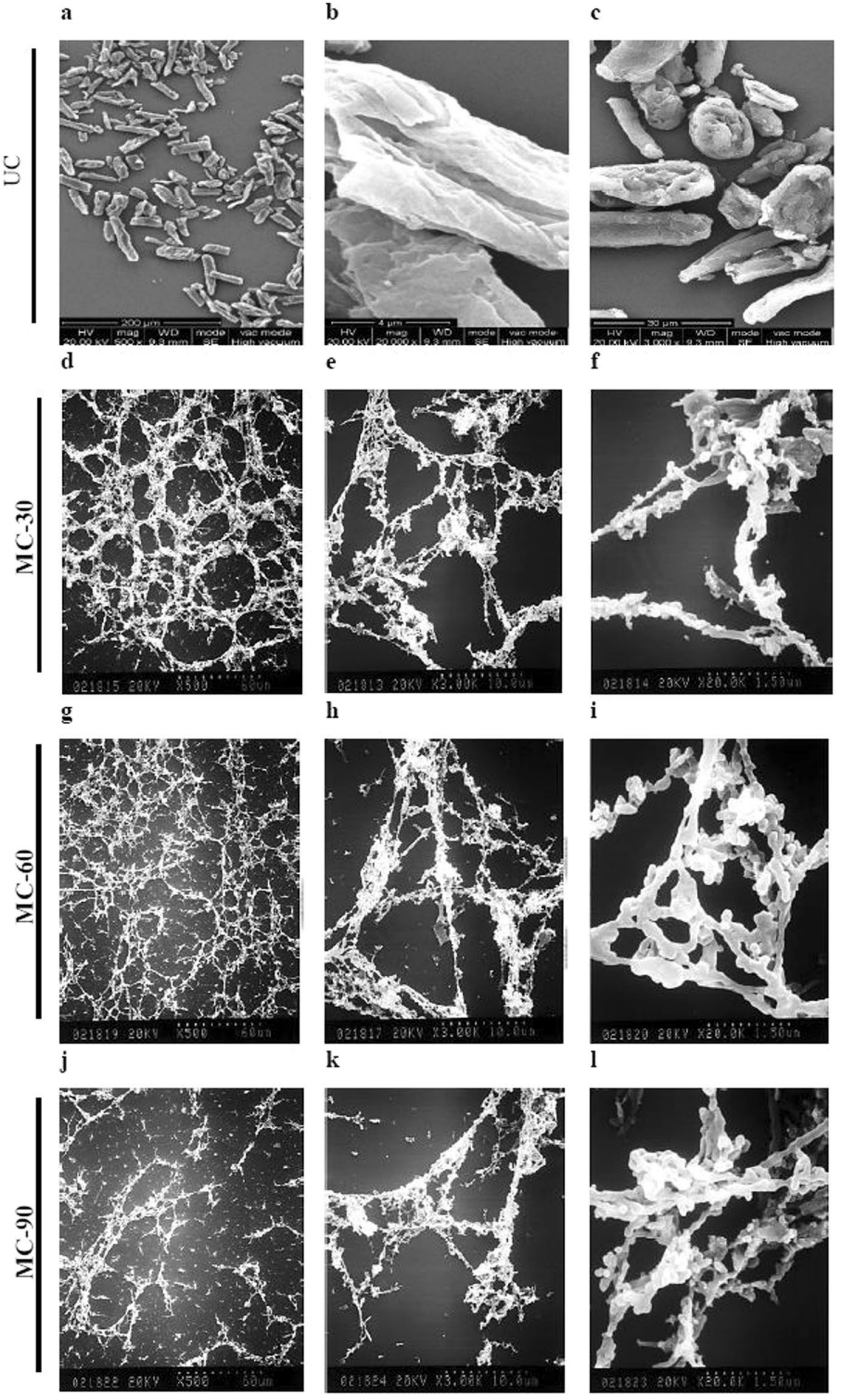
Representative scanning electron photomicrograph of cellulose at different magnifications before and after media milling. Cellulose unmilled (UC) (**a**–**c**), and milled (MC) for 30 min (**d**–**f**), 60 min (**g**–**i**) and 90 min (**j**–**l**).

Figure 3 shows SEM photomicrographs of cellulose unmilled (a–c) and milled (d–l) for different time durations. The surface of UC was found to be layered and smooth due to presence of some non-fibrous components such as lignin, waxes, pectin and oil^21^. On the other hand, MC displayed a filiform network structure completely different from the granular and integrated structure of UC. This could certainly be ascribed to frictional shear caused by media beads at the surface layers of the cellulose during milling^22^. This physical and mechanical damage induces fibrillations by breaking down hydrogen bonds, leading to transverse cleavage of cellulose fibers along the longitudinal axis. This converted the granular cellulose particles to highly entangled nanofibers. However, after prolonged milling to 90 min (Fig. 3j–l), the cellulose was completely converted into agglomerated fibers, probably due to solvent drying during gold coating under vacuum, or van der Waals interactions between the particles^23^.

### Physico-chemical properties

Table 1 represents the comparative physico-chemical properties of milled (MC) and unmilled (UC) cellulose. Intrinsic viscosities (η) were determined for UC and MC samples by extrapolation of the linear regression for c = 0. The η_i_ of dispersed cellulose decreased from 165.64 to 77.28 mL/g with increased milling time. The reduction in size of cellulose particles in MC-90 also reduced both the average molecular weight and degree of polymerization (DP) by 1.9 times. This agrees with previous reports finding the destruction of the crystalline structure of MC by high shearing force, resulting in the decline of molecular weight and DP of cellulose^24^.

**Table 1:** Physico-chemical properties of unmilled (UC) and media-milled cellulose (MC) after 30, 60 and 90 min of milling.

	UC	MC-30	MC-60	MC-90
pH	6.49	6.17	6.04	5.97
Bulk Density (g/mL)	1.41 ± 0.01^a^	1.36 ± 0.01^b^	1.35 ± 0.01^b^	1.32 ± 0.05^c^
WHC (mL/g)	3.44 ± 0.03^d^	24.99 ± 0.07^a^	19.32 ± 0.61^b^	14.03 ± 0.14^c^
Swelling (mL/g)	2.95 ± 0.07^d^	26.55 ± 0.04^a^	20.60 ± 0.85^b^	15.51 ± 0.64^c^
Specific surface area (m^2^/g)	0.27 ± 0.00^d^	5.65 ± 0.19^c^	8.94 ± 0.43^b^	9.93 ± 0.44^a^
Intrinsic viscosity (mL/g)	165.64 ± 3.46^a^	148.95 ± 2.07^b^	82.11 ± 4.56^c^	77.28 ± 3.88^c^
Molecular weight (kDa)	135.28−138.6^a^	124.25−126.32^b^	73.44−78.46^c^	70.03−74.33^c^
Degree of polymerization (DP)	2082.40−2134.81^a^	1912.59−1944.47^b^	1130.56−1207.69^c^	1077.94−1144.26^c^

Values in the same line with different lower case letters (a–d) are significantly different by Duncan’s new multiple-range test (P < 0.05).

Since the bulk density of cellulose in food is important for determining fecal transit time and excretion^1^, we measured changes in the bulk density of MC. The data showed a reduction of 3.55% in bulk density of MC-30 and a further reduction of 6.38% in MC-90, possibly due to disruption of the crystallinity of cellulose by shear forces^14^. Concurrently, the surface area of MC-90 increased by 36.78-fold (9.93 m^2^/g) compared to UC (0.27 m^2^/g). This increase in specific surface area could induce changes in the other correlated physico-chemical properties, such as water holding and swelling capacity.

After media milling for 30 minutes, the water-holding capacity (WHC) and swelling capacity increased by 7.3 and 9 times, respectively. As specified by earlier studies, swelling capacity can influence the fermentation of dietary fiber, depending on availability to microbial degradation in the colon. In contrast, hydration properties partly determine the fate of dietary fibre (DF) in the digestive tract, which accounts for fecal bulking^1^. As water holding ability is associated with the number and nature of water-binding sites^18^, exposing more water binding sites to surrounding water molecules by milling might lead to enhanced WHC and swelling capacity in MC^25^. Our results for MC-30 (Table 1) are in line with earlier reports claiming that bulk density in cellulose is directly proportional to particle size and inversely to the porosity and surface area^25,26^. However, extending the milling time from 60 and 90 min reduced WHC and swelling capacity, which is counter to the propositions of previous reports^19,25^. In this regard, we propose that WHC measurement by centrifugation method accounts for bound and trapped water. The amount of bound water depends on the chemical composition, while the trapped water is associated with structure of fiber^18^. Thus, although media milling was believed to release more hydroxyl groups of cellulose, the reduction in particle size through physico-mechanical shear subsequently destroyed the water binding sites and fiber structure of cellulose, which influenced the WHC and swelling capacity. Similar results have also been reported in a previous study on sugarcane bagasse by Sangnark and Noomhorm^27^.

### Bile acid binding capacity

**Figure 4:**
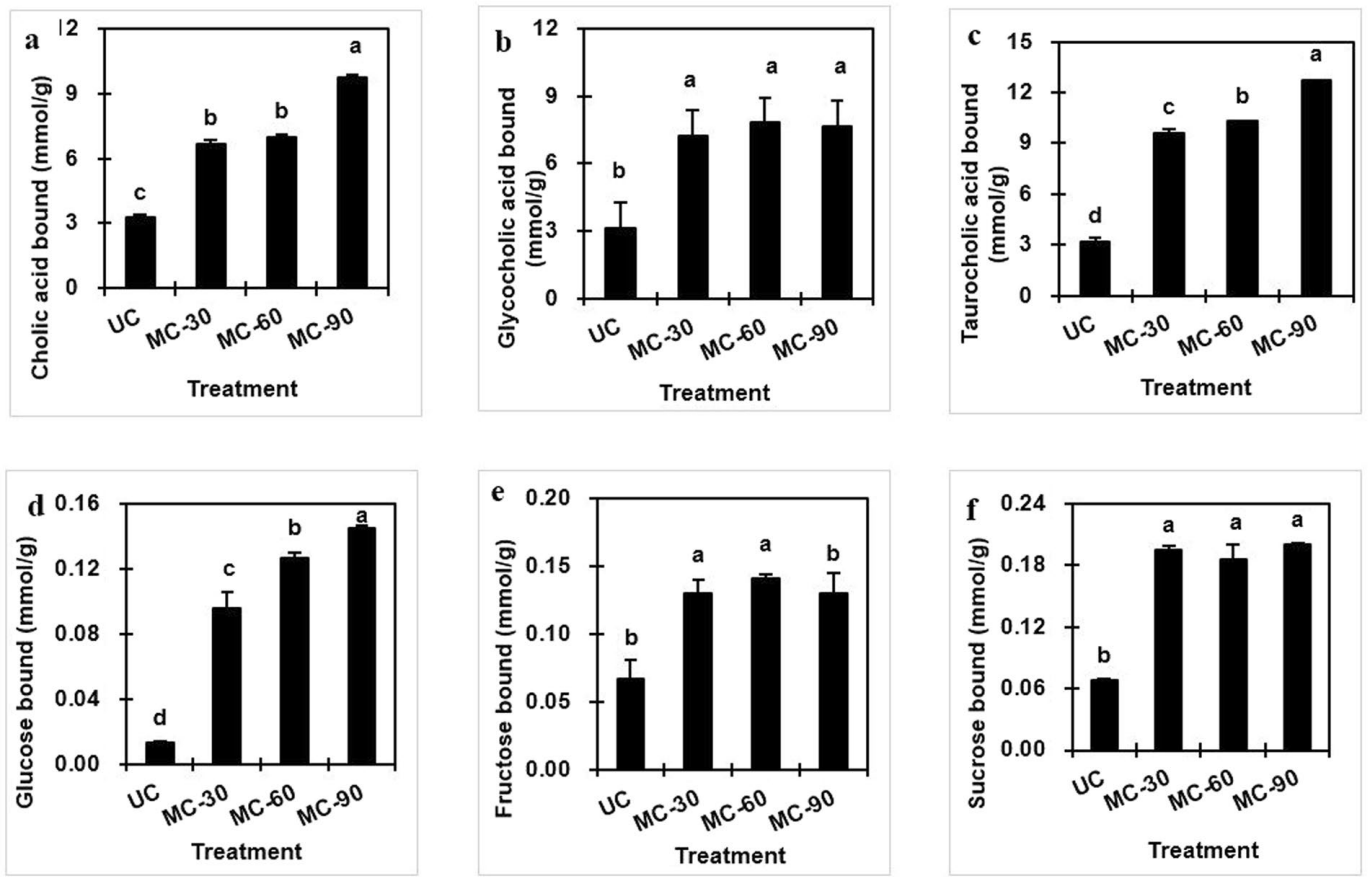
Bile acid (cholic, glycocholic and taurocholic acid) (**a**–**c**) and sugar (glucose, fructose and sucrose) binding capacity (**d**–**f**) of cellulose unmilled (UC) and media milled (MC) for 30, 60 and 90 min. Means with different letters in each panel are significantly different (P < 0.05).

Prior to secretion of any bile acids, liver cells conjugate them with one of two amino acids, glycine or taurine. The binding ability of cholic acid (CA), a primary bile acid and its glycine and taurine conjugated forms (glycocholic acid (GCA), and taurocholic acid (TCA)) was assayed for UC and MC using cholestyramine (a cholesterol-lowering, bile acid binding drug) as positive control. Although cholestyramine showed a maximum binding capacity for all three bile acids, data revealed that the MC’s reduced particle size caused an increase in the bile acid binding capacity of cellulose. As shown in Fig. 4a, MC bound more CA than UC (3.281 ± 0.107 μmol/g), and the binding capacity increased with milling time. Specifically, the CA bound by MC-90 (9.714 ± 0.169 μmol/g) was significantly higher than MC-60 (7.003 ± 0.098 μmol/g) and MC-30 (6.686 ± 0.169 μmol/g). There was a similar pattern for the bile acid conjugates, GCA and TCA (Fig. 4b,c). Binding capacities of MC-90 for GCA (7.80 ± 0.113 μmol/g) and TCA (12.747 ± 0.009 mol/g) were improved nearly 2–3 fold over UC.

Although the literatures suggest that DF may influence bile acids and cholesterol metabolism to help preventing diseases such as coronary heart disease and colorectal cancer^3^, the actual *in vivo* mechanism is still poorly understood^28,29^. It is well known that bile acids are an amphipathic detergent, capable of binding hydrophobic as well as hydrophilic molecules^30^. Reports have described a reduction in bile salt binding with decrease in cereal particle size^31^. In contrast, our results and some other studies postulate that milling exposes more hydrophilic cellulose groups due to degradation of its crystalline structure, which might improve bile acid binding capacity through interaction of cellulose and bile acids^30,32^. Furthermore, the lower pH of MC than UC observed in this study (Table 1) could have favored the bile acid binding capacity of cellulose^33^.

### Sugar binding capacity

DF has been demonstrated to exhibit a beneficial effect on the regulation of postprandial blood sugar and insulin levels^2,34^; hence, we assessed the impact of milling on the sugar binding capacity of UC and MC. The data (Fig. 4d–f) suggest that binding capacity of all three sugars were higher for MC than UC. This may be because the reduced particle size increased the surface area and porosity, leading to absorption of more carbohydrate molecules. The glucose binding capacity was improved by extending milling time, and the amount of glucose bound by MC-90 was 10-fold (0.014 to 0.14 mmol/g) higher than UC. Though no similar trend was observed in the binding capacities of three carbohydrates, the fructose and sucrose bound to MC-30 were also found to be 2.3 and 2.7- fold higher, respectively, than UC; but prolonged milling was not found to have significant effect (Fig. 4).

Moreover, the WHC of cellulose and the solubility of carbohydrates in water also seem to play a key role in the carbohydrate binding capacity of cellulose. The ability of DF to bind sugars hinders the diffusion of sugars and controls their concentration in the small intestine^2^. Fructose is considered the most hyper-triglyceridemic sugar in humans and high intake of sucrose accounts for cancerous proliferation of colon cells and insulin resistance^35^. The high binding capacity of MC for these sugars might help inhibit the colon cell proliferation, enhance insulin sensitivity and control blood triglyceride and glucose.

### Fermentability and SCFA production

The fermentability of MC was monitored by measuring changes in pH and production of SCFAs, such as acetate, propionate and butyrate. These SCFAs are very important for the colonic epithelium, colonic and intracellular pH, cell volume, and other functions associated with ion transports^36^.

**Figure 5:**
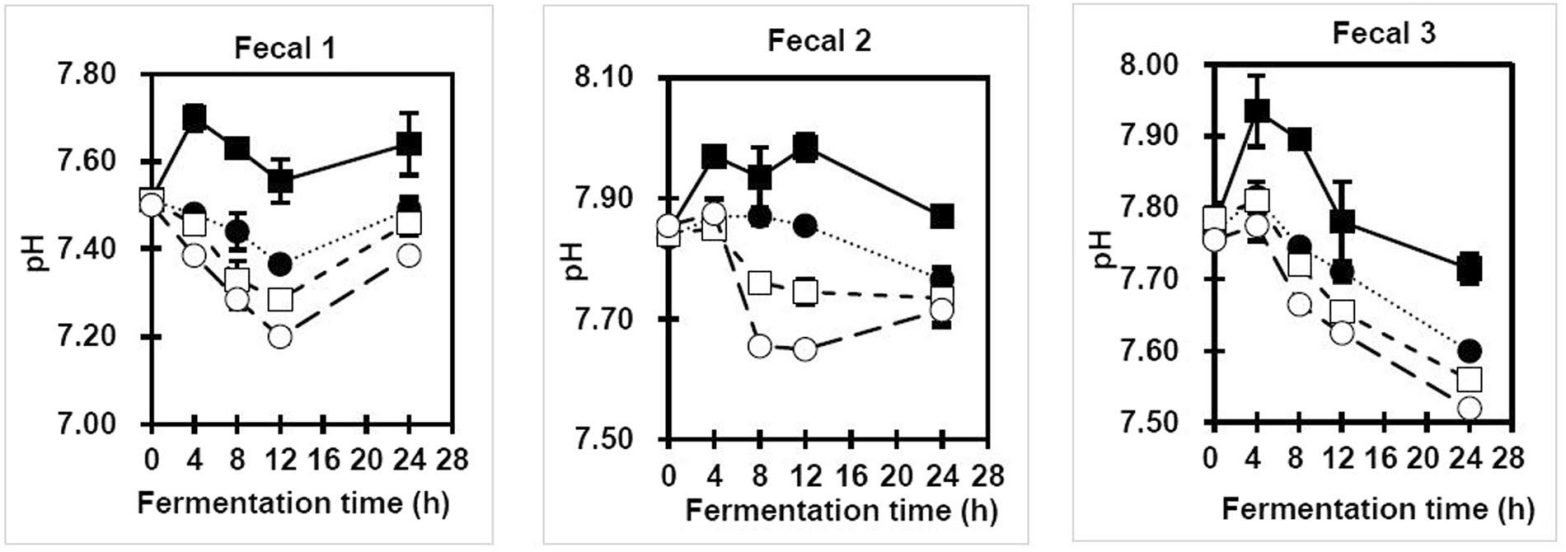
Changes in pH value of media during *in vitro* fermentation using microbiota from 3 human fecal samples. Unmilled (UC)(closed square) and milled cellulose (MC) for 30 (closed circle), 60 (open square) and 90 min (open circle).

The pH value, as an index of fiber fermentation, can be indirectly used to indicate SCFA production^37^. Lactose, as a positive control group, showed the lowest pH value during 24 h of fermentation and it was used as a marker to ensure the metabolically active status of colonic bacteria over the entire incubation time (data not shown). UC, on the other hand, was found to be the least fermentable substrate. As depicted in Fig. 5, among the MC-30, MC-60, and MC-90, the pH values of media from 3 fecal samples increased in the order of MC-90 < MC-60 < MC-30 during the fermentation. The lowest pH values were observed at 8 and 12 h of fermentation, which later increased until the end of the fermentation process. This slight increase in pH might be due to the production of different metabolites in the media, such as ammonia^38^. A lower intestinal pH value is claimed to reduce the conversion rate of primary to secondary bile acids and lower their carcinogenic potential^39^. Moreover, it also might protect against pathogenic bacteria, stimulate the growth of the beneficial *Bifidobacteria* and *Lactobacillus* generas^40^ and promote the absorption of minerals, such as calcium and magnesium^41^.

**Figure 6:**
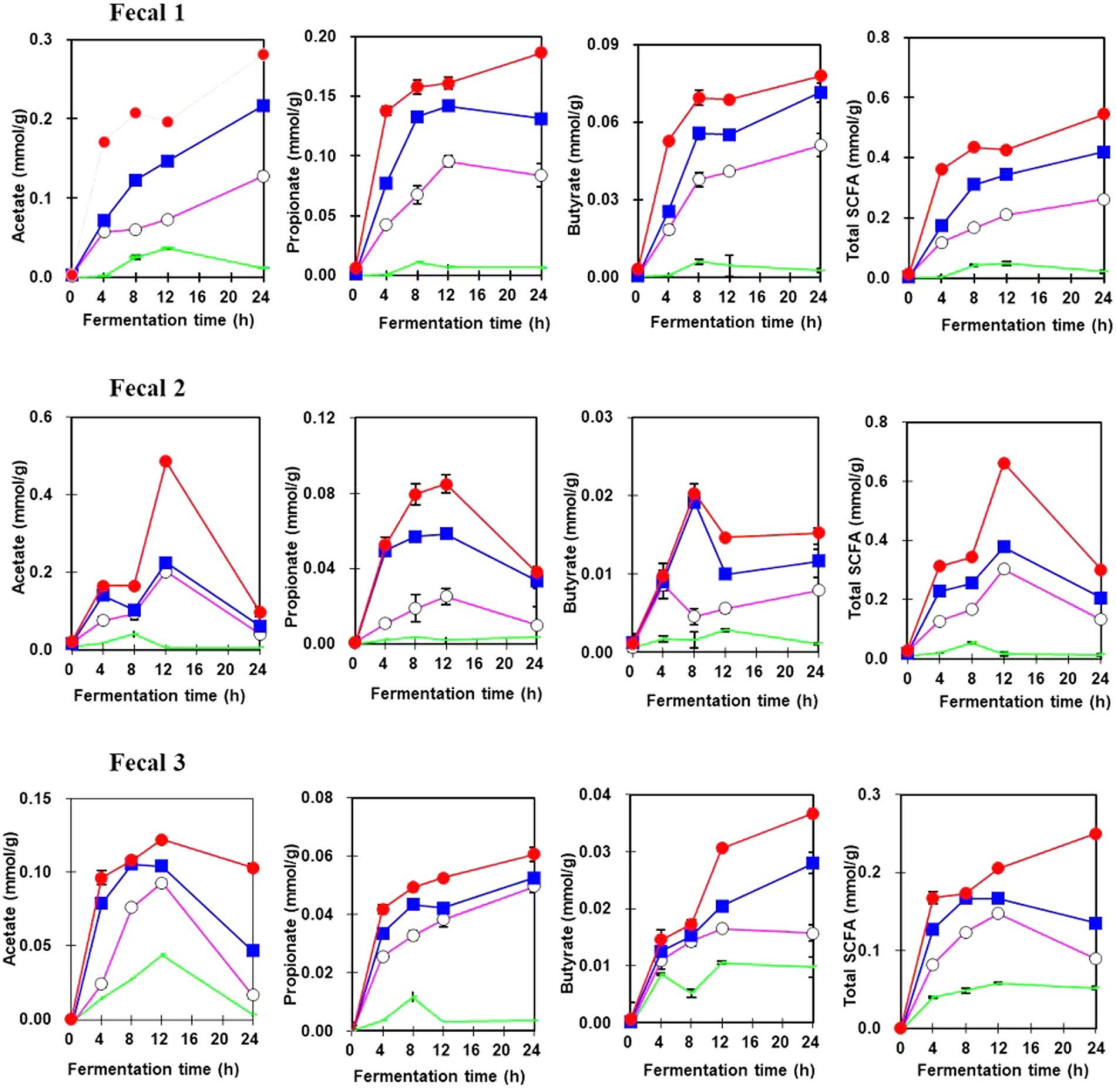
Production of SCFA (acetate, propionate, and butyrate) by *in vitro* batch fermentation. Fermentation of unmilled (no marker) and media milled cellulose for 30 (open circle), 60 (closed square) and 90 min (closed circle) using three different human fecal samples.

Since it is difficult to measure the rate of SCFA production *in-vivo* due to inaccessibility of the human colon, *in-vitro* batch fermentation systems with the micro-flora of fresh fecal materials were used in this study to mimic the human digestive tract for predicting the physiological effects of cellulose consumption and its influence on fecal bulk. Figure 6 demonstrates the fermentation of milled cellulose (MC) using the microflora from 3 fecal samples. The production of short chain fatty acids like acetate, propionate and butyrate was found to be higher during fermentation of MC compared to UC. This might be ascribed to pretreatments during media milling, which decreased molecular weight and increased the surface area of MC, thereby easing fermentation by colonic microflora^40^. Of all three fecal samples, acetate has the highest production, followed by propionate and then butyrate. Although there was a trend for the milled samples in fermenting cellulose (MC-30 < MC-60 < MC-90), no regular pattern in production of SCFAs was found over the entire fermentation time among all 3 fecal samples. The acetate concentrations of MC increased until 24 h of fermentation in fecal 1, while it dropped after 12 h in fecal samples 2 and 3. The reduction in acetate after 12 h may be because metabolic pathways convert acetate to butyrate in the late fermentation period^42^. In contrast, the increasing acetate production by fecal 1 may account for the variations in the dominant metabolic type of butyrate produced between individuals or with variations in diet which could influence butyrate production in the large intestine^43^. Moreover, the bacterial species present in the colon use different fermentation pathways, leading to differences in the SCFA pattern generated^44^.

MC-90 also generated the highest amount of propionate (0.188 μmol/g) and butyrate (0.077 μmol/g); but consistent with acetate results, no regular trend in production of propionate amount was found over the entire fermentation time among all the samples (Fig. 6). Fermentation using fecal 1 and 3 showed highest propionate and butyrate production at 24 h but in case of fecal 2 it reduced after 12 h. As cellulose is considered to be the main contributor in propionate production^45^, breakdown of non-starch polysaccharides by milling may account for higher propionate production^38^ in MC. Moreover, Duncan *et al*.^43^ reported that 85% of butyrate is generated from acetate, whereas only 15% of butyrate is generated directly from glucose or the Embden-Meyerhof-Parnas (EMP) pathway. For this reason, the higher amount of butyrate generated from MC after 12 h might be attributed to the conversion of acetate. Enhanced production of acetate and propionate has also been reported in a similar *in-vitro* study using high molecular weight indigestible dietary fibres (dextrin, α-cyclodextrin, and dextran) without causing any alteration in colonic microbiota^46^.

Owing to its high molecular weight and crystallinity, low degradability and low solubility, UC produced the least, while MC-90 produced the most total SCFAs in all samples (Fig. 6). This indicates that MCs are better substrates for SCFA production compared to UC. Specifically, the production of total SCFA by fecal 2 was highest (0.66 μmol/g) after 12 h while for fecal 1 and 3, the maximum SCFA production (0.54 and 0.25 μmol/g respectively) was obtained after 24 h of fermentation.

With all the 3 fecal intestinal micro-biota combinations, the MC-90 showed the highest potential in SCFA production. The differences in SCFA production among samples can possibly be ascribed to differences in gastrointestinal flora between individuals; additionally, factors such as age, diet, stress, lifestyle and bacterial digestibility might also have influenced the amount and ratio of SCFA produced. Many other determinants, such as diet or climate, aging, medication (especially antibiotics), illness, stress, pH, infection, geographic location, race, socioeconomic circumstances, and lifestyle can upset this balance^20,47^. In addition to various positive and significant outcomes, this study includes a limitation that microbial species in the fecal samples used were not identified or quantified. As the production of SCFA is largely controlled by the composition of gut microbiota and bacterial cross-feeding interactions has a huge impact on the final amount of SCFA production, its identification could have allowed to assess the effect of MC on each microbial strain in fecal samples. Moreover, the research outcomes of this study provides the basis for further validation of beneficial physiological effects of MC in an *in-vivo* model.

## Conclusions

Size reduction after milling, gives cellulose characteristic properties, such as enhanced binding of water, bile, and sugar in digesta, thus offering the possibility to ameliorate various health issues. Moreover, higher SCFA production, swelling capacity and bulk density of MC give it a physiological benefit for maintaining colon health. Thus, it can be concluded that prolonged milling, particularly for 90 min (MC-90), seem to more effective than the unmilled cellulose, thus opening the possibility to find strategies for developing personalized nutrition. As a result, incorporating milled cellulose in food products can be a profitable value-added option for manufacturers to better align their products with consumer desires for healthier food.

## Methods

### Sample preparation

Microcrystalline cotton cellulose (designated as unmilled cellulose, UC) (Sigma Cellulose, Type 20) was purchased from Sigma-Aldrich Inc., St. Louis, MO., U.S.A. As described in our previous study^48^, the milled cellulose was prepared using a media mill (Minipur, Netzsch-Feinmahltechnik GmbH, Germany) with a driving motor of 0.94 kW. Figure 1 is a schematic diagram of the milling process. Media beads (0.8 mm yttria-stabilized tetragonal zirconia, YTZ) were loaded in the milling chamber (200 mL) at 70% v/v filling ratio. A 4% wt. suspension of UC (20 g) was prepared using a high speed blender (Polytron, PT 3100, Kinematica, Lucerne, Switzerland) for 10 min with 500 mL deionized water (dH_2_O) at 3000 rpm. The UC suspension was loaded into a jacket-cooled tank (Fig. 1) and then fed at a flow rate of 250 mL/min into the milling chamber by a circulating pump. Suspension temperature was maintained below 30 °C and agitation speed was set at 3600 rpm. Milling was continued for 90 min and samples were removed at 30-, 60- and 90-min and labelled as MC-30, 60 and 90 for further analyses. The zirconium contamination in the milled cellulose due to wearing of beads was tested and it not detected (data not shown).

### Particle size distribution (PSD)

PSD of samples was determined by laser diffraction particle size analyzer (LS 230, Beckman Coulter, CA., U.S.A.) with detection range of 0.04–2000 µm. The instrument was calibrated with dH_2_O. All samples were diluted 100 times and vortexed for 10 s. Average diameters (both in volume and in number) of particles were obtained by LS 230 software. All measurements were made in triplicate and average data are reported.

### Morphology

The change in morphology of cellulose particles after media milling was examined by scanning electron microscope (SEM) (Hitachi S-800, Hitachi Co. Ltd., Tokyo, Japan). Cellulose suspensions were diluted 10 times and then distributed on top of the glass. After sputter-coating with gold, SEM micrographs were observed at 20 kV.

### Intrinsic viscosity

Since cellulose is insoluble in LiCl/DMAc solutions at room temperature, we used a method involving swelling followed by solvent exchange^49^. Cellulose powder (4 g) was suspended overnight in 100 mL dH_2_O, and ethanol was added to replace water. The ethanol-cellulose mixture was vacuum evaporated (90 rpm, 30 °C, 15 min) and further air-dried (25 °C). Lithium chloride was dissolved in N, N-dimethyl acetamide (DMAc) at 40 °C to prepare a 9% (w/w) solution, and the swollen cellulose (1 g) sample was dissolved in 20 mL 9% LiCl/DMAc by stirring at room temperature for 3–5 h. Dissolved cellulose was diluted to five different concentrations and the intrinsic viscosities were determined using a suspended level Ubbelohde viscometer at 25.0 ± 0.1 °C. The viscosity-average molecular weight was determined from intrinsic viscosity of the polymer following Chamberlain *et al*.^50^. The relation between the intrinsic viscosity, molar mass (M), and degree of polymerization (DP) was derived according to the empirical Mark-Houwink equation^51,52^.

### Intrinsic bulk density (volumetric method)

Freeze dried MC (1 g) was quantified by dH_2_O in a 50 mL weighted volumetric flask and degassed by ultrasonic cleaner for 1 min at 40 kHz. The intrinsic bulk density (D_c_) was calculated as:

1 $${{\rm{D}}}_{{\rm{c}}}=\frac{{{\rm{W}}}_{{\rm{c}}}}{{{\rm{V}}}_{{\rm{c}}}},\,(\frac{{\rm{g}}}{{\rm{mL}}})$$

where W_c_ is the weight of cellulose (specified as g), and V_c_ is the corresponding volume evaluated by

2 $${{\rm{V}}}_{{\rm{c}}}={\rm{50}}-\frac{{{\rm{W}}}_{{\rm{w}}}}{{{\rm{D}}}_{{\rm{w}}}},\,(\mathrm{mL})$$

Here, W_w_ is the weight of water, and D_w_ is the density of water at room temperature.

### Specific surface area

Specific surface area (a) was calculated by applying the number percentage (n_i_%) and volume percentage (v_i_%) of particles determined by laser diffraction particle size analyzer (LS230)

3 $${\rm{a}}=\sum _{{\rm{i}}}{{\rm{n}}}_{{\rm{i}}}(\pi {{\rm{d}}}_{{\rm{i}}}^{{\rm{2}}})=\pi {\rm{n}}\sum _{{\rm{i}}}{{\rm{d}}}_{{\rm{i}}}^{{\rm{2}}}{{\rm{n}}}_{{\rm{i}}} \% =\frac{{\rm{6w}}}{\rho }\frac{\sum _{{\rm{i}}}{{\rm{d}}}_{{\rm{i}}}^{{\rm{2}}}{{\rm{n}}}_{{\rm{i}}} \% }{\sum _{{\rm{i}}}{{\rm{d}}}_{{\rm{i}}}^{{\rm{3}}}{{\rm{n}}}_{{\rm{i}}} \% }$$

where d_i_ is the diameter of particles, n is total number of particles ($=\sum _{{\rm{i}}}{{\rm{n}}}_{{\rm{i}}}$), w is dry weight of cellulose in (g), and *ρ*is the density of cellulose in (g/mL).

### Water holding capacity (WHC) and swelling capacity

WHC was assayed by the method developed by Robertson *et al*.^18^ with some modifications. Cellulose suspension (0.2 g in 5 mL) was centrifuged at 1000 *g*, 15 °C for 15 min in a 15 mL centrifuge tube. Pellets were dried at 105 °C for 24 h and the weight of water held per gram of dry material was measured. Swelling capacity (volume of water swollen per gram of dry material) was obtained by deducing the volume of supernatant released after centrifugation from the previously added volume^6^.

### Bile acid binding capacity (BABC)

The BABC was determined by the method of Kahlon and Woodruff^53^ with some modifications. 5 mL of 720 μmol/L bile acid solution (0.1 M pH 7.0 phosphate buffer) was added to 2.5 mL cellulosic suspension and incubated at 37 °C for 2 h in a shaker bath. The mixture was centrifuged at 1000 *g* and 15 °C for 15 min, and the supernatant was filtered through a 0.45 μm membrane filter. The filtrate was assayed using a commercial bile acid (TBA) kit (Randox Laboratories Ltd). Cholestyramine, a bile acid binding anionic resin was used as a positive control to verify the enzyme activity.

### Sugar binding capacity (SBC)

SBC was determined following the method of Chau *et al*.^25^ with some modifications. A 2.5 mL sample (0.1 g solid content) was mixed with 5 mL (10 mmol/L) of sugar solution (glucose, fructose and sucrose) in a centrifuge tube at 37 °C for 6 h. The mixture was centrifuged at 2000 *g* for 15 min. The supernatant was filtered through a 0.45 μm filter, and 200 μL of filtrates from each sugar were used to measure total sugar concentration using the phenol-sulfuric assay^7^.

### *In vitro* fermentation and production of short chain fatty acid (SCFA)

The batch-culture technique utilizing fresh healthy fecal microflora, from non-methanogenic humans consuming a normal diet was employed to assess the fermentability with 2 replicate samples^54^. This study was conducted between the year of 2008 and 2009 in accordance with Taiwanese ethical guidelines for human research and all subjects provided informed consent. The subjects confirmed they had not received antibiotics for at least 6 months and not suffered from indigestion within the previous week. As the digesta enter the large intestine about 12 h after ingestion, the batch model system used in this study was measured from 0–24 h with the expectation that between 12 and 24 h would be the time points most relevant to the large intestine.

Fresh human feces were loaded into a phosphate buffer solution at 37 °C and homogenized by stomacher for 2 min. Filtrated fecal samples were diluted (1:6 w/v) by phosphate buffer solution containing reducing solution. Then 2.5 mL of cellulose suspension was mixed with 8 mL of buffer and incubated for overnight hydration at 4 °C. Substrate hydration and preparation of carbonate-phosphate buffer containing trace elements, reducing agent and resazurin (as a redox indicator) were conducted by following the method of Lebet *et al*.^55^. Lactose samples and blanks were selected as fermentation controls. Then 3 mL of the fecal inoculum was mixed with hydrated substrate and placed into anaerobic jars with anaerobic indicator strips at 37 °C. Microbial activity was terminated by the addition of 0.1 mL saturated HgCl_2_ at 0, 4, 8, 12 and 24 h. The pH values of samples were measured and aliquots were frozen at −20 °C for SCFA analysis.

Measurement of SCFA concentrations was modified from Lebet *et al*.^55^. Samples were thawed and centrifuged at 5000 *g*, 4 °C for 10 min. 5 mL supernatant was mixed with 0.5 mL H_3_PO_4_ solution (50 mg/mL, containing 10 mg/mL HgCl_2_) and the acidified supernatants were filtered through 0.45 μm filter. 4-methyl-valeric acid was added as an internal standard. Acetate, propionate, and butyrate were determined by a gas chromatograph (Hewlett Packard model 6890, Palo Alto, CA, USA) with a 007-FFAP (50 m × 0.32 mm ID × 0.25 mm film) column at an oven temperature of 110 °C for 6 min, then raised at 50 °C/min to 160 °C. Injector temperature was 250 °C, with split of 1:40, and FID detector temperature was 255 °C. Flow rates for helium, hydrogen, and air were 1.5, 45, and 400 mL/min, respectively. A standard mixture containing six SCFA at the expected concentration and 4-methyl-valeric acid as an internal standard was used for quantification.

### Statistical analysis

Statistical analyses were conducted using a SAS version 9.1.3 (SAS Institute, Cary, NC, USA). Data were reported as means ± SD of three separate experiments performed in triplicate. Statistical comparisons were conducted by one-way analysis of variance (ANOVA), followed by a Duncan’s multiple comparison test. Differences were considered as statistically significant when the P values were below 0.05 (P < 0.05).

### Data availability

The data generated during and/or analysed during the current study are available from the corresponding author on reasonable request.
